# Community mental health status six months after the Sewol ferry disaster in Ansan, Korea

**DOI:** 10.4178/epih/e2015046

**Published:** 2015-10-23

**Authors:** Hee Jung Yang, Hae Kwan Cheong, Bo Youl Choi, Min-Ho Shin, Hyeon Woo Yim, Dong-Hyun Kim, Gawon Kim, Soon Young Lee

**Affiliations:** 1 Department of Preventive Medicine and Public Health, Ajou University School of Medicine, Suwon, Korea; 2 Department of Social and Preventive Medicine, Sungkyunkwan University School of Medicine, Suwon, Korea; 3 Department of Preventive Medicine, Hanyang University College of Medicine, Seoul, Korea; 4 Department of Preventive Medicine, Chonnam National University Medical School, Gwangju, Korea; 5 Department of Preventive Medicine, The Catholic University College of Medicine, Seoul, Korea; 6 Department of Social and Preventive Medicine, Hallym University College of Medicine, Chuncheon, Korea

**Keywords:** Disasters, Community surveys, Mental health, Screening, Posttraumatic stress

## Abstract

**OBJECTIVES:**

The disaster of the Sewol ferry that sank at sea off Korea’s southern coast of the Yellow Sea on April 16, 2014 was a tragedy that brought grief and despair to the whole country. The aim of this study was to evaluate the mental health effects of this disaster on the community of Ansan, where most victims and survivors resided.

**METHODS:**

The self-administered questionnaire survey was conducted 4 to 6 months after the accident using the Korean Community Health Survey system, an annual nationwide cross-sectional survey. Subjects were 7,076 adults (≥19 years) living in two victimized communities in Ansan, four control communities from Gyeonggi-do, Jindo and Haenam near the accident site. Depression, stress, somatic symptoms, anxiety, and suicidal ideation were measured using the Center for Epidemiologic Studies-Depression Scale, Brief Encounter Psychosocial Instrument, Patient Health Questionnaire-15, and Generalized Anxiety Disorder 7-Item Scale, respectively.

**RESULTS:**

The depression rate among the respondents from Ansan was 11.8%, and 18.4% reported suicidal ideation. Prevalence of other psychiatric disturbances was also higher compared with the other areas. A multiple logistic regression analysis revealed significantly higher odds ratios (ORs) in depression (1.66; 95% confidence interval [CI], 1.36 to 2.04), stress (1.37; 95% CI, 1.10 to 1.71), somatic symptoms (1.31; 95% CI, 1.08 to 1.58), anxiety (1.82; 95% CI, 1.39 to 2.39), and suicidal ideation (1.33; 95% CI, 1.13 to 1.56) compared with Gyeonggi-do. In contrast, the accident areas of Jindo and Haenam showed the lowest prevalence and ORs.

**CONCLUSIONS:**

Residents in the victimized area of Ansan had a significantly higher prevalence of psychiatric disturbances than in the control communities.

## INTRODUCTION

On April 16, 2014, the Sewol ferry with 476 passengers sank at sea off South Korea (hereafter Korea)’s southern coast of the Yellow Sea. The number of dead and missing from this accident was 304, of whom 250 were high school students on a field trip. This disaster was a tremendous shock to the entire country, leaving indelible emotional scars especially in the community of Ansan, where most victims and survivors as well as their families resided.

Many studies have reported that such national disasters can trigger adverse outcomes not only among victims and their families, relatives, and friends, but also among a broad spectrum of the population exposed to the disaster situation [1-3], including voluntary support workers and community residents who indirectly experienced the disaster through the media [4,5]. In particular, human disasters tend to occur in places with high concentrations of human and material resources, thus involving a high death toll and affecting the entire society [6]. For the purpose of this study, it was hypothesized that accidents involving specific local residents and students would have a greater effect on the community concerned.

The effect of accidents and disasters on health status manifests in various forms, not only psychiatric symptoms such as posttraumatic stress disorder and depression, but also somatic symptoms, substance abuse, interpersonal relationship difficulties, and loss of social network [7]. The mental health response to disaster is an important research area, as shown by a large number of studies on the 9/11 terrorist attack. Higher prevalence of posttraumatic stress was found in New York compared with Washington and other metropolitan areas [4], and the closer to the terrorist attack site the higher the prevalence [1]. In Korea, in the wake of the Hebei Spirit oil spill in 2007, a high prevalence of depression, stress, and suicidal ideation was observed among the residents of the victimized area [8]. A one year follow-up study of community health status in the affected areas reported high burdens of disease through mental disorders [9].

Population groups exposed to a disaster undergo a generally observed process of showing strong emotional and psychological reactions for up to one year, and begin to recover after the anniversary reaction [10]. In this process, adequate interventions can shorten the recovery period or mitigate psychological harm, whereas health problems can persist without such interventions. Although the Trauma Center built in Ansan after the disaster has provided counseling and monitoring for the bereaved families and related individuals [11], the level of support and research provided for community residents is not up to the magnitude of shock suffered by them. The effects of emotional and psychological harm suffered by the community residents as a whole should be analyzed to establish adequate disaster mental health response plan.

The purpose of this study was to determine the effect of a disaster on a local community by evaluating the mental health status of community residents in terms of depression, stress, somatic symptoms, anxiety, and suicidal ideation. Evaluation took place six months after the disaster using a survey-based approach comparing different geographical areas.

## MATERIALS AND METHODS

### Study population

The study was conducted in 2014 using the national community health survey system. The Korean Community Health Survey (KCHS) is an annual national cross-sectional survey system based on a standardized questionnaire carried out by the Korea Centers for Disease Control and Prevention at the community level to produce regional health indicators that serve as a basis for establishing community health plans [12]. The KCHS conducted in 254 local districts, and the target population in each area was about 900 adult residents (≥19 years). The KCHS used a two-stage sampling process. The first stage was to apply a probability proportional to size sampling strategy (to select primary sampling units) and the second stage was to apply systematic sampling (selecting households). The KCHS collects various information on demographic and socioeconomic characteristics, health-related problems and past medical histories, administered by trained interviewers as face-to-face interviews.

**Figure 1.**
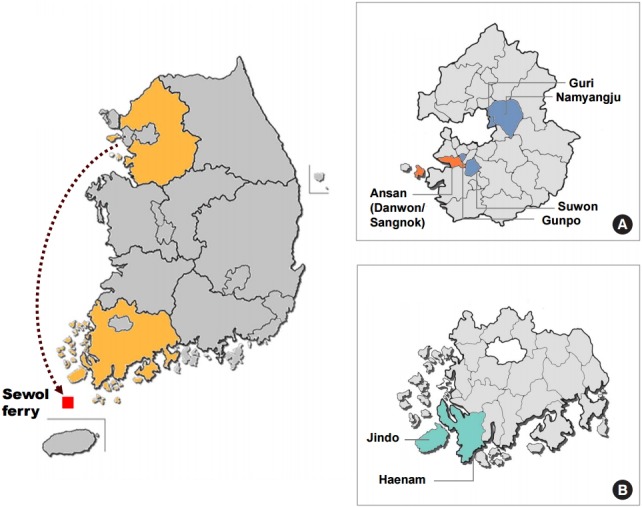
Map of the study area and sampling locations in (A) Gyeonggi-do, (B) Jeollanam-do.

Eight survey locations were selected: two victimized communities in Ansan (Danwon and Sangnok), four control communities in Gyeonggi-do (Paldal in Suwon, Gunpo, Guri, and Namyangju), Jindo and Haenam near the accident site involved in salvage works. A supplementary self-administered questionnaire was distributed to the residents of the communities who completed the KCHS after receiving a separate informed consent from each respondent (Figure 1).

The survey was conducted from August 16 to October 31. The total sample was 7,310 participants: 918 and 923 from Danwon and Sangnok in Ansan city, 916 and 917 from Paldal (Suwon) and Gunpo in the southern Gyeonggi province, 912 and 917 from Guri and Namyangju of the northern Gyeonggi province, and 911 and 896 from Jindo and Haenam, respectively. Of the 7,310, a total of 7,153 individuals (97.9%) signed the informed consent form. Another 77 respondents that could not be identified due to errors in survey numbers resulted in 7,076 participants (96.8%) that were included in this study. This study was approved by the institutional review board (IRB) of Ajou University (IRB no. SBR-SUR-14-252), and participants received an explanation pointing to their rights concerning refraining from providing responses and withdrawing from the study at any time.

### Measurements

The data used for the current study were those concerning the general characteristics included in the KCHS survey and the mental health screening results from the mental health status questionnaire (Appendix 1). General characteristics collected in the KCHS were location, sex, age, education level, and monthly household income. Depression, stress, somatic symptoms, anxiety, and suicidal ideation were evaluated using self-report screening tools.

The Center for Epidemiologic Studies-Depression Scale (CES-D) is a non-diagnostic screening measure of depression consisting of 20 items that are responded to on a 4-point scale. Score of 21 or higher is epidemiologically defined as depression [13]. The Korean version of the CES-D [14] was used in this study.

To measure stress, the Brief Encounter Psychosocial Instrument [15] (the Korean version translated by Lim et al. [16]) was used. Participants respond to 5 items on a 5-point scale, and average scores up to 1.6, 1.6 to 2.8, and higher than 2.8 are defined as low-level, moderate-level, and high-level stress, respectively.

The Patient Health Questionnaire-15 (PHQ-15) is a measure of somatic symptoms based on the Patient Health Questionnaire (PHQ) domains of somatic, anxiety, and depressive symptoms as simplified by Kroenke et al. [17]. It is used for evaluating somatic symptoms and estimating the degree of somatization. The measure consists of 15 items that are responded to on a 3-point scale. Scores of 5 to 9, 10 to 14, and 15 or higher are indicative of low-level, moderate-level, and high-level severity of somatic symptoms, respectively. In this study, the Korean version of the PHQ-15 translated by Han et al. [18] was used.

The Generalized Anxiety Disorder 7-Item Scale (GAD-7) was used to measure generalized anxiety disorder. Developed as a general anxiety measurement tool, it is used as a comprehensive screening tool for anxiety disorders, such as panic disorder, social phobia, and posttraumatic stress disorder [19]. The measure consists of 7 items that are responded to on a 4-point scale. Scores of 5 to 9, 10 to 14, and 15 or higher indicate low-level, moderate-level, and high-level severity of symptoms, respectively.

Suicidal ideation was evaluated based on an affirmative or negative answer to the question “Have you ever thought of taking your own life?” that is included in the KCHS in every evennumbered year.

### Statistical analysis

Sociodemographic characteristics of the participants such as sex and age distributions and education and income levels in the three study areas were processed on an interval scale. The mental health screening results for each area are expressed in percentages and average scores, and the inter-area comparison of mental health status is presented in standardized prevalence rates. The standardized prevalence rates were calculated using the direct standardization method for sex and age on the basis of the standard population defined in the Census conducted in 2005 by Statistics Korea. Regional differences in prevalence were estimated using Pearson’s chi-square test. A multiple logistic regression analysis was performed to control for sociodemographic variables, and the estimated mental health prevalence rates in victimized and accident areas are presented as odds ratios (OR) with 95% confidence intervals (CI) relative to those in the areas used as a control. Statistical analysis was performed using SPSS version 24.0 (IBM Corp., Armonk, NY, USA), and the values were considered statistically significant at p<0.05 for a two-tailed test.

## RESULTS

The mean age of the subjects (n=7,076) was 49.7 years, with males accounting for 44.9% of the sample. The rural areas of Jindo and Haenam had a higher mean age than the urban areas in Ansan and control cities, as well as higher percentages of female residents and lower education and income levels (Table 1).

Prevalence in Ansan for depression, moderate-to-severe stress, somatic symptoms, anxiety, and suicidal ideation were 11.4%, 8.8%, 11.9%, 5.9%, and 17.7% (crude rate), respectively. The highest levels among the three areas and the regional differences proved statistically significant (p<0.05) (Table 2). After age and sex standardization as well, Ansan’s prevalence rates were highest. Jindo and Haenam showed the lowest post-standardization prevalence rates.

**Figure 2.**
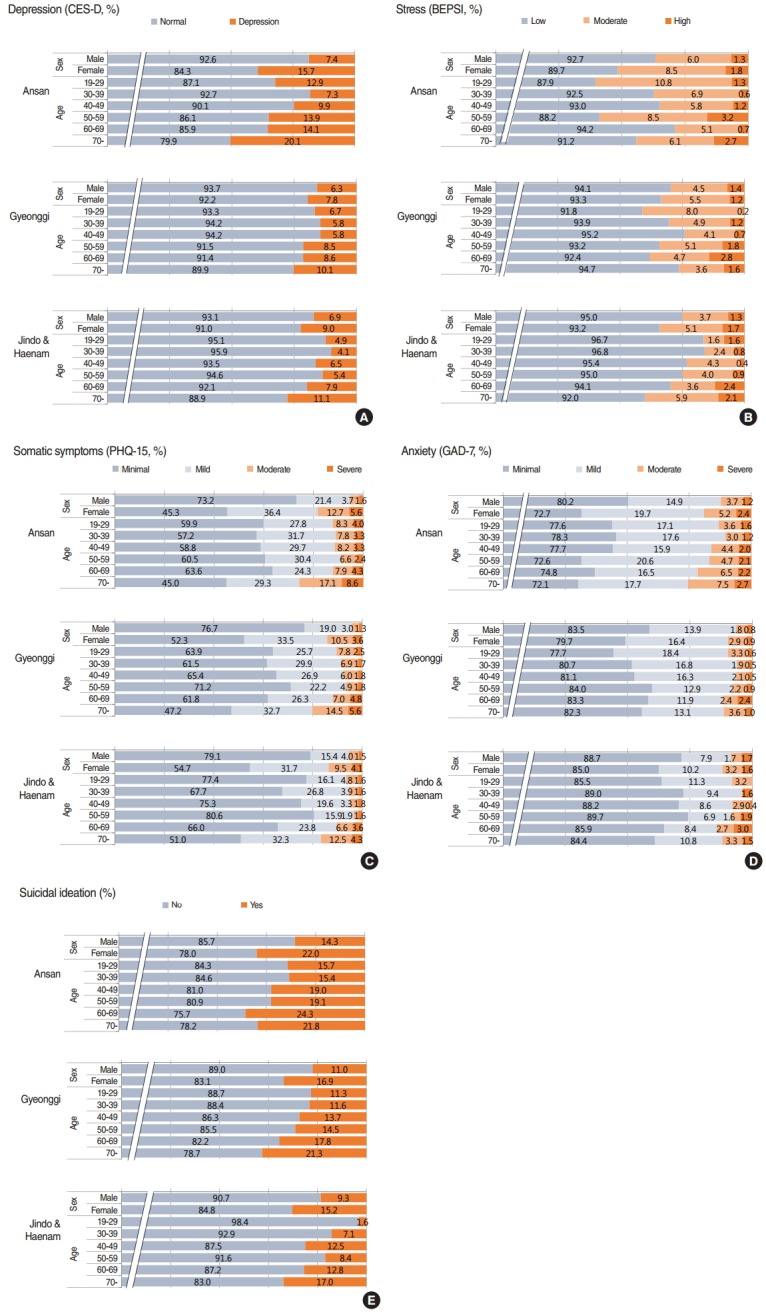
Regional distribution of mental health screening results (A: depression, B: stress, C: somatic symptoms, D: anxiety, E: suicidal ideation) presented by sex/age group. CES-D, Center for Epidemiologic Studies Depression Scale; BEPSI, Brief Encounter Psychosocial Instrument; PHQ-15, Patient Health Questionnaire-15; GAD-7, Generalized Anxiety Disorder 7-Item Scale.

The results for different sex and age groups confirmed the overall tendency of higher prevalence rates in Ansan than the other two areas for all age groups (Figure 2). Females showed a higher prevalence of all psychiatric symptoms irrespective of area. In general, upward trends in depression, anxiety, and suicidal ideation were observed with increasing age. The prevalence of stress in the young and middle-aged groups in urban areas (Ansan and Gyeonggi) were generally high compared with Jindo and Haenam.

A multiple logistic regression analysis was performed to adjust for age, sex, education, and income, followed by inter-area ORs for psychiatric symptoms (Table 3). Compared with the control area (Gyeonggi), Ansan showed significantly higher adjusted ORs for depression (1.66; 95% CI, 1.36 to 2.04), stress (1.37; 95% CI, 1.10 to 1.71), somatic symptoms (1.31; 95% CI, 1.08 to 1.58), anxiety (1.82; 95% CI, 1.39 to 2.39), and suicidal ideation (1.33; 95% CI, 1.13 to 1.56) compared with the other areas. In contrast, Jindo and Haenam showed low ORs in all domains.

## DISCUSSION

In this study, the short-term to mid-term effects of the Sewol ferry disaster on the affected community were evaluated by conducting a survey on the mental health status of residents 4 to 6 months after the disaster. Compared to the control area (Gyeonggi), the Ansan area most heavily affected by the disaster exhibited higher prevalence rates of the psychiatric symptoms assessed, and similarly high ORs were yielded by the multiple logistic regression analysis adjusting for sociodemographic features. Furthermore, Jindo and Haenam near the accident site showed the lowest prevalence of psychiatric symptoms, contrary to expectation, presumably due to its character as a rural area.

The relevance of the Sewol ferry disaster as it relates to the significantly higher prevalence of psychiatric symptoms in the Ansan area may be estimated by investigating existing data. The 2009 KCHS measured depression rates using the CES-D, and suicidal ideation is assessed every other year. In 2009, the age-standardized depression rates in Danwon and Sangnok of the Ansan area were 4.3% and 4.8%, respectively. The rates for Gunpo, Paldal (Suwon), Guri, and Namyangju in Gyeonggi were 6.2%, 8.3%, 7.1%, and 5.9%, respectively; and for Jindo and Haenam rates were 1.7% and 3.5%, respectively [20]. The 2014 depression data gathered in this survey demonstrated considerably higher depression rates in the Ansan area (11.8%) as well as in Jindo and Haenam (8.1%). With a time gap of five years, this increase cannot be exclusively attributed to the effects of the Sewol ferry disaster, but it may be assumed to have certain effects, given that no significant changes took place in the control area (6.9% vs. 5.9 to 8.3%).

With regard to suicidal ideation, after age standardization the affirmative answer accounted for 9.6% (Danwon) and 17.2% (Sangnok) in Ansan in 2013, 7.2% (Gunpo), 10.6% (Paldal/Suwon), 8.1% (Guri), and 13.5%, (Namyangju) in Gyeonggi, and 3.3% and 4.9% in Jindo and Haenam, respectively [20]. This indicates that in one year’s time suicidal ideation increased in all surveyed communities except for Namyangju, with Danwon in Ansan demonstrating the highest increase of 7.6%. However, the higher baseline value in the Ansan area compared with other areas should be taken into account. This may be interpreted as a higher vulnerability of already affected subjects to the effects of the disaster [21].

As indicators allowing for time-series comparison, experience of depressive symptoms and perceived stress questions, one item each, are annually assessed in the KCHS. In Ansan, the age-standardized rate of depressive symptoms increased from 9.8% in 2013 to 13.0% in 2014 in Danwon, and decreased from 12.7% to 8.1% in Sangnok, although higher than the average of 7.0% in Gyeonggi [20]. Danwon showed the highest depression rate of the 254 communities across the country. This is consistent with our survey results using the CES-D and indicative of the effects of the Sewol ferry disaster. However, it should be taken into account that the depression rate in Ansan was also high in 2013 and over the 75% quantile. Perceived stress slightly decreased from 33.3% to 31.7% in Danwon and from 35.6% to 26.2% in Sangnok, without showing any noticeable differences from the Gyeonggi average of 30.2% (95% CI, 29.7% to 30.7%) [20]. These findings deviate slightly from what was found in this study for stress.

Somatic symptoms and anxiety were also measured in this study. The ORs in the Ansan area were higher than in Gyeonggi. This result is consistent with the literature that indicates unexpected disaster gives rise to anxiety through uncertainty about danger [22], and triggers various nonspecific symptoms inexplicable with physical disorders [23]. Even though anxiety is a normal reaction to disaster in the short term, a prolonged period of anxiety can develop into a chronic state of anxiety and trigger psychosomatic problems [24].

The age-dependent prevalence of depression shown in this study is similar to the study of Oh et al. [25] conducted in 2009, in which the CES-D results from the community health survey were analyzed and mapped by age. Subjects in their 30s showed the lowest depression prevalence, which increased with age, whereas those aged 19 to 29 showed a slightly higher depression rate than people in their 30s. The effect of the disaster on children and adolescents could not be assessed because this study only targeted adults. This aspect should be investigated in future research, given that early detection of and intervention for psychological injury in children and adolescents still in the formative years of their lives are of vital importance [26].

Most of the mental health research on large-scale disasters has been conducted focusing on survivors or bereaved families, and only a small number of studies have examined the effects of disaster on the general population at the community level. There are a few community-based studies in relation to the ocean contamination by the 2007 Hebei Spirit oil spill, but most participants were individuals directly involved through cleanup works or residents who suffered financial losses [8,9,27]. In other countries, too, most studies targeted eyewitnesses or residents within the areas affected by natural disasters, such as typhoons and earthquakes [5,28]. In contrast, the Sewol ferry disaster did not take place in a physical space of residence of the victims, and residents experienced the accident only indirectly via media or acquaintances. It is also distinct from other disasters in that the main victims were adolescents from the communities concerned, thus bringing a tremendous shock and severe emotional and psychological trauma to community members of all ages. Taking into account such distinct features and the implications of this study, comprehensive mental health monitoring and interventions are necessary for the community members.

Psychological and emotional tension caused by a disaster escalates the collective stress of the whole community. Fears of other disasters and worries about impending crises are ignited. Such a changed atmosphere and loss of human and material resources caused by the disaster can create long-term stress [29]. Although psychological damage may mitigate over time, if left uncared for or complicated by other negative factors long-term problems can develop. This study was conducted 4 to 6 months after the disaster, and a Taiwanese study conducted at the same point in time similarly reported symptoms of posttraumatic stress disorder and severe depression after a major disaster [28]. Such symptoms have also been reported as much as seven years after the disaster [5,29]. A follow-up survey in relation to the 9/11 terrorist attack noted that persisting long-term psychological symptoms were more associated with absence of timely interventions than with direct exposure to the disaster [2]. Therefore, interventions should be administered timely.

One of the limitations of this study is the selection of control group. Four regions in Gyeonggi, the province where Ansan is located, were selected as control communities to minimize regional deviations, but even after adjusting for sociodemographic features there were inter-region deviations. Although Ansan showed a higher prevalence rate in 2014, the health index related to mental health status showed an elevated level in a prior survey. Jindo and Haenam had low prevalence rates in the present and prior surveys but their geographical features are distinct from Gyeonggi, which did not allow for direct comparison with the control communities. Moreover, the Sewol ferry disaster was a national tragedy that was reported in real time via media, leaving strong emotional imprints in all Koreans. As such, there may have been an underestimation of the effects of the disaster on the accident region.

Another limitation was the lack of data and the impossibility of time-series observation. However, meaningful observations could be made using different forms of variables and past data. As for depression and suicidal ideation, different reactions of respondents between a face-to-face interview and self-administered questionnaire should also be taken into account, because sensitive questions may have been answered more honestly in the latter such that underestimation of past prevalence rates cannot be ruled out.

As a third limitation, it should be pointed out that individuals directly involved in the disaster, such as bereaved families and relatives, could not be targeted for sampling due to the nature of the community-based sampling system of this study. Furthermore, items evaluating the direct relevance of the Sewol ferry disaster were excluded from the questionnaire to forestall prejudices related to the disaster. This did not allow for the application of detailed risk classification models in accordance with the degree of exposure. Nor could we apply the survey instrument for posttraumatic stress disorder because most community samples did not directly experience the disaster. However, some studies showed that residents who had vicariously experienced a national disaster developed posttraumatic stress disorder [3]. It is therefore considered necessary to develop a screening tool for evaluating disaster-related stress in the general population.

Despite these limitations, various aspects of mental health were evaluated using screening tools with recognized efficiencies. A salient feature of this study is its representativeness, in which systematically extracted samples were used from the existing community health survey system. It is also significant that this study could evaluate the mental health status of communities within six months after the disaster. Given the results of this cross-sectional survey, it is considered necessary to provide adequate interventions to actively help residents of victimized communities recover their mental health status. A follow-up study to observe the change and recovery process in the affected communities will be significant from both academic and social perspectives.

## Figures and Tables

**Table 1.** Sociodemographic characteristics of the study population (n = 7,076)

Variables	Ansan (n=1,773)		Gyeonggi (n=3,507)		Jindo & Haenam (n=1,796)		p-value^2^
	n^1^	%	n	%	n	%	
Sex							
Male	821	46.3	1,591	45.4	762	42.4	0.046
Female	952	53.7	1,916	54.6	1,034	57.6	
Age (yr)							
19-29	306	17.3	512	14.6	62	3.5	<0.001
30-39	338	19.1	775	22.1	127	7.1	
40-49	500	28.2	818	23.3	280	15.6	
50-59	342	19.3	674	19.2	322	17.9	
60-69	140	7.9	422	12.0	339	18.9	
≥ 70	147	8.3	306	8.7	666	37.1	
Education							
Middle school or lower	359	20.2	688	19.7	1,155	64.4	<0.001
High school	854	48.2	1389	39.7	412	23.0	
College or higher	560	31.6	1,424	40.7	227	12.7	
Income (10^4^ Korean won/mo)							
≤ 100	163	9.2	302	8.9	795	44.4	<0.001
101-200	229	12.9	433	12.8	412	23.0	
201-300	398	22.4	657	19.5	222	12.4	
301-400	368	20.8	677	20.0	143	8.0	
≥ 401	615	34.7	1,308	38.7	217	12.1	

1 Actual (unweighted) sample size. Missing values on education (n=8); income (n=137).

2 p-values were calculated by chi-square tests.

**Table 2.** Prevalence of psychological distress detected by screening tests

Screening instruments	Ansan (n=1,773)		Gyeonggi (n=3,507)		Jindo & Haenam (n=1,796)		p-value^2^
	n^1^	%	n	%	n	%	
Depression (CES-D)							
Normal	1,515	88.2	3,179	92.8	1,608	91.9	<0.001
Depression	203	11.8	245	7.2	141	8.1	
Score (mean±SD)	10.54±9.00		8.45±8.02		8.14±8.57		
Age-sex standardized rate (depression)^3^		11.4		6.9		5.8	
Stress (BEPSI)							
Low	1,608	91.1	3,272	93.6	1,681	94.0	0.001
Moderate	130	7.4	177	5.1	80	4.5	
High	28	1.6	45	1.3	28	1.6	
Score (mean±SD)	0.81±0.67		0.72±0.62		0.51±0.66		
Age-sex standardized rate (moderate-high)		8.8		6.5		4.4	
Somatic symptoms (PHQ-15)							
Minimal	1,012	58.3	2,190	63.4	1,148	65.0	0.001
Low	511	29.4	930	26.9	439	24.8	
Moderate	148	8.5	246	7.1	127	7.2	
High	65	3.7	89	2.6	53	3.0	
Score (mean±SD)	5.36±4.38		4.87±4.06		4.81±4.10		
Age-sex standardized rate (moderate-high)		11.9		9.4		6.4	
Anxiety (GAD-7)							
None	1,343	76.1	2,839	81.4	1,548	86.6	<0.001
Mild	309	17.5	532	15.3	165	9.2	
Moderate	79	4.5	85	2.4	46	2.6	
Severe	33	1.9	30	0.9	29	1.6	
Score (mean±SD)	2.87±3.69		2.28±3.10		1.72±3.36		
Age-sex standardized rate (moderate-severe)		5.9		3.2		3.2	
Suicidal ideation							
No	1,445	81.6	3,004	85.8	1,565	87.3	
Yes	326	18.4	498	14.2	228	12.7	<0.001
Age-sex standardized rate (“yes”)		17.7		13.5		8.5	

CES-D, Center for Epidemiologic Studies Depression Scale; BEPSI, Brief Encounter Psychosocial Instrument; PHQ-15, Patient Health Questionnaire-15; GAD-7, Generalized Anxiety Disorder 7-Item Scale; SD, standard deviation.

1 Missing values on depressive disorder (n=185); stress (n=27); somatic symptoms (n=118); anxiety (n=38); suicidal ideation (n=10).

2 p-values were calculated by chi-square tests.

3 Age-sex standardized rates were calculated using direct standardization with the 2005 Census performed by the Statistics Korea as the reference.

**Table 3.** Adjusted odds ratios and 95% confidence interval for the logistic regression of psychological distress by region

	Depression (CES-D)	Stress (BEPSI, moderate-high)	Somatic symptoms (PHQ-15, moderate-high)	Anxiety (GAD-7, moderate-severe)	Suicidal ideation
Ansan	1.66 (1.36, 2.04)^***^	1.37 (1.10, 1.71)^**^	1.31 (1.08, 1.58)^**^	1.82 (1.39, 2.39)^***^	1.33 (1.13, 1.56)^***^
Gyeonggi	1.00 (reference)	1.00 (reference)	1.00 (reference)	1.00 (reference)	1.00 (reference)
Jindo & Haenam	0.54 (0.42, 0.69)^***^	0.62 (0.48, 0.82)^**^	0.50 (0.40, 0.63)^***^	0.66 (0.47, 0.92)^*^	0.52 (0.43, 0.63)^***^

Adjusted for sex, age, education, income. CES-D, Center for Epidemiologic Studies Depression Scale; BEPSI, Brief Encounter Psychosocial Instrument; PHQ-15, Patient Health Questionnaire-15; GAD-7, Generalized Anxiety Disorder 7-Item Scale.

* p<0.05,

** p<0.01,

*** p<0.001.
