# Supplementary material for: Community mental health status six months after the Sewol ferry disaster in Ansan, Korea
Source: Epidemiol Health. 2015 Oct 23;37:e2015046. doi: 10.4178/epih/e2015046 (PMC4835706; doi:10.4178/epih/e2015046)
Supplement: Supplementary file 1 [file epih-37-e2015046-supplementary.pdf]

Community mental health status six months after the Sewol ferry disaster in Ansan

안산시 세월호 사건 6개월 후 지역사회 정신건강 수준

양희정, 정해관, 최보율, 신민호, 임현우, 김동현, 김가원, 이순영

Hee Jung Yang[1], Hae Kwan Cheong[2], Bo Yul Choi[3], Min Ho Shin[4], Hyeon Woo Yim[5], Dong Hyun Kim[6], Gawon Kim[1], Soon Young Lee[1]

Department of Preventive Medicine and Public Health, Ajou University School of Medicine, Suwon, Korea

Department of Social and Preventive Medicine, Sungkyunkwan University School of Medicine, Suwon, Korea

Department of Preventive Medicine, College of Medicine, Hanyang University, Seoul, Korea.

Department of Preventive Medicine, Chonnam National University Medical School, Gwangju, Korea.

Department of Preventive Medicine, College of Medicine, The Catholic University, Seoul, Korea

Department of Preventive Medicine, Kyungpook National University School of Medicine, Taegu, Korea

---

Corresponding author: Soon Young Lee, MD. PhD

206 World cup-ro, Yeongtong-gu, Suwon 443-721, Korea

Tel: +82-31-219-5301, Fax: +82-31-219-5084

E-mail: solee@ajou.ac.kr

Community mental health status six months after the Sewol ferry disaster in Ansan, Korea

한국 안산시 세월호 사건 6개월 후 지역사회 정신건강 수준

ABSTRACT

Objectives: 2014년 4월 16일 한국의 서해 남단에서 발생한 세월호 침몰 사고는 한국 사회 전체에 엄청난 슬픔을 안긴 사건이었으며, 특히 대부분의 희생자가 발생한 안산시 지역사회가 받은 충격은 더욱 컸다. 본 연구는 이 재난 상황이 지역사회의 정신건강에 미친 영향을 평가하기 위해 실시하였다.

Methods: 매년 실시하는 국가적 단면 조사인 2014년 지역사회건강조사를 기반으로 사고 이후 4-6개월 시점에 조사를 실시하였다. 피해 지역(안산) 및 대조 지역(안산을 제외한 경기도의 4개 지역), 사고 지역(진도-해남) 및 을 선정, 19세 이상 성인 7,076명을 대상으로 자기기입식 설문조사를 시행하였으며, The Center for Epidemiologic Studies-Depression Scale (CES-D), Brief Encounter Psychosocial Instrument (BEPSI), Patient Health Questionnaire-15 (PHQ-15), Generalized anxiety disorder 7-item scale (GAD-7) 을 사용하여 우울, 스트레스, 신체 증상, 불안 및 자살생각에 대해 평가하였다.

Results: 안산 대상자의 11.8%가 우울군으로 분류되었으며, 18.4%가 자살생각을 호소하였고, 그 밖의 유병률도 타 지역에 비해 높았다. 다중 로지스틱 회귀 분석 결과 비교 지역에 비해 안산의 우울증상률, 중등도 이상의 스트레스, 신체증상, 불안, 자살생각에 대한 오즈비가 각각 1.66(95% CI 1.36-2.04), 1.37(95% CI 1.10-1.71), 1.31(95% CI 1.08-1.58), 1.82(95% CI 1.39-2.39), 1.33(95% CI 1.13-1.56)으로 유의하게 높았다. 반면 사고 지역인 진도-해남은 도농 지역으로 나머지 지역과 구별되는 지역적 특성을 가지고 있어, 유병률 및 오즈비가 경기 및 안산 지역보다 낮았다.

Conclusion: 재난 피해 지역인 안산시 주민의 정신건강 유병수준이 대조 지역에 비해 유의하게 높았다.

Key words: disasters, community surveys, mental health, screening, post-traumatic

INTRODUCTION

2014년 4월 16일 한국의 서해 남단에서 476명의 승객을 태운 여객선 세월호가 침몰하였다. 이 사고로 인해 304명의 사망자 및 실종자가 발생하였으며, 이 중 250명이 한 고등학교의 수학여행 차 탑승한 학생이었다. 한국 사회 전체에 엄청난 충격을 안긴 사건이었으나, 특히 희생자 및 생존자와 그 가족이 주로 거주하고 있는 경기도 안산시의 지역사회에 미친 영향들이 컸다.

이러한 국가적 재난 사고 후 직접적인 피해를 받은 당사자와 친인척은 물론이고, 재난 상황에 노출된 광범위한 인구 집단에게 건강 영향이 발생할 수 있다는 것은 여러 연구에서 보고된 바 있다 [1-3]. 희생자의 주변인이나 자원봉사자 등은 물론이고, 미디어 등을 통하여 재난을 간접적으로 경험한 지역사회 주민들도 영향권에 포함된다 [4, 5]. 특히 인적 재난은 인구와 물자가 집중된 곳에 빈번히 발생하는 경향이 있어 인명피해의 규모가 크며, 부주의와 관리 소홀에 대한 우려로 인해 사회 전반에 미치는 영향이 크다 [6]. 특히 본 사건과 같이 특정 지역민들, 그리고 학생에게 큰 희생이 발생한 경우 해당 지역사회에 미치는 영향은 더욱 클 것으로 예상되었다.

재난 및 재해로 인한 건강 영향은 외상후 스트레스 장애, 우울, 불안, 공황장애 등 잘 알려진 정신과적 문제에서부터 비특이적인 신체증상, 기존 질병의 악화 및 새로운 증상 발생 등의 신체적 증상까지 다양하게 나타나며, 수면, 알콜, 약물, 흡연 등의 의존도가 증가하거나, 대인관계의 문제나 스트레스 등과 같은 일상생활의 만성적 문제를 발생시키고, 사회적 결속력을 저하시킴으로서 사회 네트워크의 기능상실을 유발하는 등 다양한 측면에서 건강에 영향을 미치는 것으로 알려져 있다 [7]. 이 중 특히 재난으로 인한 정신적 영향은 중요한 영역으로, 대표적으로 9/11 테러와 관련하여 많은 연구가 이루어진 바 있다. 워싱턴 및 다른 대도시에 비해 사건이 발생한 뉴욕시에서 [4], 지역 내에서는 테러가 발생한 지역에 가까울수록 높은 외상후 스트레스가 관찰되었다 [1]. 국내에서는 2007년 허베이 스피리트 호 유류유출 사고의 피해지역 주민에서 우울, 스트레스와 자살충동이 높게 나타났고 [8], 사고 후 1년간 관찰 결과 해당 지역의 정신질환으로 인한 질병부담이 높았던 것으로 보고된 바 있다 [9].

일반적으로 재난에 노출된 집단은 이후 1년까지 강한 정서적 변동을 겪은 후, 기념일 반응을 기점으로 회복되는 일련의 과정을 겪는 것으로 보고되어 있다 [10]. 이 과정에서 적절한 개입을 통

해 회복 과정을 앞당기거나 정신적 피해를 완화할 수 있으며, 적절한 개입이 이루어지지 않을 경우 건강 문제가 오래 지속될 수 있다. 재난 이후 안산 트라우마센터를 건립하여 유족 및 관계자들에 대한 상담 및 모니터링을 제공하고 있으나 [11], 지역사회가 받았을 것으로 예상되는 충격에 비해 일반 지역사회 주민에 대한 지원이나 연구는 부족한 실정이다. 향후 지역사회의 회복 및 심리적 모니터링 대책을 마련하기 위해서는 일반 지역사회 주민에 대한 정신적 피해의 영향을 파악할 필요가 있다.

본 연구에서는 일부 지역사회를 표본으로 한 조사를 통해 세월호 사건 약 6개월 후 지역사회 주민의 정신건강을 우울, 스트레스, 신체 증상, 불안, 자살생각의 측면에서 평가하고, 지역 간의 차이를 관찰함으로써 재난이 특정 지역사회에 미친 영향을 파악하고자 하였다.

MATERIALS AND METHODS

Study Population

본 연구는 지역사회 기반으로 매년 실시하는 국가적 단면 조사인 지역사회건강조사 체계를 기반으로 2014년 시행하였다. 지역사회건강조사는 지역보건의료계획 수립 및 평가를 위한 지역 간 건강지표를 생산하기 위해 질병관리본부가 주관하여 2008년부터 전국을 대상으로 실시하고 있는 표준화된 조사 체계이다 [12]. 각 지역에 거주하는 만 19세 이상의 모든 주민을 모집단으로 전국 254개의 지역 보건소 단위별로 약 900명의 표본이 조사될 수 있도록 확률비례통계추출을 통해 표본지점을 선정하고, 선정된 표본지점에서 계통추출법에 의해 조사가구를 선정, 해당 가구의 만 19세 이상 성인 모두를 대상으로 전문 조사자가 대면 조사 방식을 통해 조사하는 방식으로 이루어진다.

본 연구에서는 세월호 사건 피해의 중심지인 안산에 해당하는 2개 지역 (안산 단원, 안산 상록)과, 대조군으로 경기도 내의 4개 지역(수원 팔달, 군포, 구리, 남양주), 침몰 지역과 인접하여 구조 작업이 주로 이루어진 전라남도 진도 및 해남의 2개 지역을 포함한 총 8개조사 단위의 지역을 선정, 해당 지역의 지역사회건강조사 설문을 완료한 사람을 대상으로 별도 동의를 얻어 자기 기입식 추

가 설문지 형태로 조사를 실시하였다[figure 1]. 조사는 8월 16일부터 10월 31일까지 실시되었다. 안산 지역인 안산 단원, 안산 상록의 지역사회건강조사 완료자는 각 918명, 923명이었고, 대조군인 경기도 내의 다른 지역인 경기 남부의 수원시 팔달구, 군포시는 916명, 917명, 경기 북부의 구리시, 남양주시는 각 912명, 917명, 사고지역인 진도, 해남은 각 911명, 896명으로 총 7,310명이 추가 조사 대상자가 되었다. 이 중 총 7,153명(97.9%)가 동의하였으며, 이 중 조사번호 누락 및 오류로 인해 지역사회건강조사와 연계가 불가능한 77건을 제외한 7,076명 (96.8%)이 분석 대상에 포함되었다. 본 연구는 아주대학교 기관윤리심의위원회의 승인을 받았으며 (IRB NO: SBR-SUR-14-252), 조사 전 충분한 설명과 함께 동의서를 받고 원하지 않을 경우 답변하지 않거나 조사를 중단할 수 있음을 설명하였다.

Measurements

본 연구에 사용된 자료는 지역사회건강조사에 포함된 일반적인 특성에 관한 자료와 정신건강조사 설문지[appendix]에 포함된 정신건강 영역의 선별검사 결과 자료이다. 두 자료는 개인식별정보가 포함되지 않은 일련번호를 통해 연계하여 분석하였다. 지역사회건강조사에서 수집한 일반적 특성에는 거주 지역, 성별, 연령, 학력, 월평균 가계소득이 포함되어 있으며, 정신건강조사 설문은 자기기입식으로 평가가 가능한 선별검사 도구를 사용하여 우울, 스트레스, 신체화 증상, 불안에 대해 평가하였으며, 자살생각에 관한 1문항을 포함하였다.

우울증을 측정한 도구인 The Center for Epidemiologic Studies-Depression Scale (CES-D)는 역학조사를 위한 가치가 인정되어 있는 도구로, 20문항의 4점 척도로 이루어진 비진단적 선별 검사이며, 역학적 연구 기반에서 합산 점수가 21점 이상일 경우 우울증이 있다고 정의한다 [13]. 본 조사에서는 조 등이 번역한 한국판 [14]을 사용하였다.

스트레스를 평가하는 도구로는 Brief Encounter Psychosocial Instrument (BEPSI) [15] 을 사용하였으며, 필요에 따라 선택하여 사용할 수 있는 지역사회건강조사의 잠재적 선택 문항에 포함되어 있다. 5점 척도의 5문항의 평균이 1.6이하일 경우 저스트레스, 2.8-1.6점인 경우 중등도 스트레스,

2.8점 이상인 경우 고스트레스로 정의하며, 임 등이 번역한 한국판 [16] 을 사용하였다.

Patient Health Questionnaire-15 (PHQ-15) 는 Patient Health Questionnaire (PHQ)의 3가지 영역 (somatic, anxiety, depression) 중 신체 증상 분야를 Kroenke 등이 간추린 도구로서 [17], 주관적 신체증상을 평가하는 도구이다. 신체 증상의 정도를 평가하고, 신체화 증상(somatization)을 추정하는 데 활용 가능하며, 국내에서는 특히 우울증상이 신체증상으로서 나타나는 비중이 큰 것으로 알려져 있다. 3점 척도의 15문항으로 이루어져 있고, 5-9점일 경우 경도, 10-14점일 경우 중등도, 15점 이상일 경우 고도의 신체 증상이 있다고 평가한다. 한 등이 번역한 한국판을 사용하였다 [18].

불안을 평가하는 도구로 사용한 Generalized anxiety disorder 7-item scale (GAD-7)은 범불안장애 평가도구로 개발되었으나 공황장애, 사회공포증, 외상 후 스트레스 장애 등을 포함한 포괄적인 불안장애의 선별이 가능한 것으로 알려져 있다 [19]. 4점 척도의 7가지 문항으로, 5-9점일 경우 경도의 불안, 10-14점일 경우 중등도의 불안, 15점 이상일 경우 고도의 불안으로 평가한다.

자살생각에 대한 문항은 지역사회건강조사에서 짝수 해마다 격년도로 조사하고 있는 것과 동일 문항을 사용하였으며, “1년 동안 죽고 싶다는 생각을 해 본 적이 있습니까?” 의 질문에 예/아니오로 답하였다.

Statistical Analysis

연구대상자의 성별, 연령, 학력, 가계소득 등 일반적 특성을 각 지역별로 제시하였다. 연령 및 소득을 포함한 모든 변수는 구간 변수로 처리하였다. 지역별 정신건강 선별검사 결과를 등급별 분율 및 평균 점수로 나타내었으며, 지역 간의 정신건강 수준을 비교하기 위해 표준화된 유병률을 제시하였다. 표준화된 유병률은 2005년 통계청 인구조사에서 정의된 표준인구를 사용하여 성별, 연령에 따라 직접표준화법으로 계산하였다. 피어슨의 카이제곱을 이용해 지역별 유병률의 차이에 대한 검정을 시행하였다. 인구사회학적 변수를 통제하기 위해 일반적 특성을 포함한 다중 로지스틱 회귀 분석을 시행하여, 대조 지역을 준거로 한 피해 지역 및 사고 지역의 정신건강 유병에 관

한 오즈비와 95% 신뢰구간을 제시하였다. 자료의 통계 분석은 SPSS version 24 프로그램을 사용하였으며, 결과의 통계적 유의성은 $p<0.05$를 기준으로 양측 검정하였다.

RESULTS

총 대상자인 7,076명의 평균 연령은 49.7세였으며, 남성이 44.9%였다. 도시 지역인 안산 및 비교지역에 비해 도농 지역인 진도-해남에서 연령층이 높았으며, 이에 따라 여성 비율과 저학력, 저소득의 비율이 높았다 [table 1].

각 지역별 선별검사 결과 안산 지역의 우울, 중등도 이상의 스트레스, 신체증상, 불안, 자살생각의 유병률은 각 11.4%, 8.8%, 11.9%, 5.9%, 17.7%(crude rate)로 3지역 중에서 가장 높았으며, 카이제곱 검정 결과 모든 선별검사에서 지역에 따라 결과 등급에 유의한 차이를 보였다($p<0.05$) [table 2]. 안산의 유병률은 연령 및 성별 표준화를 실시한 후에도 가장 높았다. 반면 사건 발생 지역인 진도-해남 지역에서 표준화 후의 유병률이 가장 낮았다.

각 지역별로 성별 및 연령별로 선별 검사 결과를 관찰한 경우에도, 대부분의 연령층에서 안산 지역의 유병률이 타 지역에 비해 높았다[figure 2]. 모든 지역, 모든 검사에서 여성의 유병률이 남성보다 높았다. 연령별로는 우울, 불안 및 자살생각의 경우 연령이 높아질수록 대체로 높아지는 추세를 보였다. 도시 지역에 속하는 안산 및 경기 지역의 청장년층의 스트레스 유병률은 진도-해남에 비해 대체로 높았다. 로지스틱 회귀 분석을 통해 다변량 분석을 시행하여 연령, 성별, 학력, 소득을 보정 후 선별검사 및 자살생각에 대한 지역 간 오즈비를 산출하였다[table 3]. 경기도 비교군에 비해 안산 지역에서 우울, 스트레스, 신체 증상, 불안, 자살생각의 보정된 오즈비가 각각 1.66(95% CI 1.36-2.04), 1.37(95% CI 1.10-1.71), 1.31(95% CI 1.08-1.58), 1.82(95% CI 1.39-2.39), 1.33(95% CI 1.13-1.56)으로 유의하게 높았다 ($p<0.05$). 반면, 진도 및 해남 지역에서는 모든 영역에서 낮은 오즈비를 보였다.

DISCUSSION

본 연구를 통해 재난의 지역사회에 대한 영향을 평가하였다. 재난 이후 약 6개월 내에 조사를 실시함으로써 중단기적인 영향을 평가하기 적절하였다. 연구 결과 경기도 대조 지역에 비해 재난의 영향을 가장 많이 받은 안산 지역에서 정신건강의 모든 영역에서 유병률이 높았고, 인구사회학적 특성을 보정한 로지스틱 회귀 분석 결과에서도 유의하게 높은 오즈비를 보였다. 단, 전후 비교가 가능하지 않아 이것이 세월호 사건의 영향이라고 단정하기는 어려웠다. 그리고, 세월호 사고 지역으로 재난의 영향을 받았을 것으로 추정했던 진도 및 해남에서 농어촌 지역이라는 고유한 특성으로 인해 모든 정신건강의 유병률이 낮게 나타났을 수 있으며, 경기도와 비교하여 유병수준이 낮다고 해서 재난 피해로 인한 정신적 영향이 없었다고 단정할 수는 없었다.

본 조사에서 나타난 안산의 높은 유병률이 세월호 사고의 영향인지 추정해 보기 위해 기존 자료를 살펴볼 필요가 있다. 2009년 지역사회건강조사에서는 CES-D를 사용하여 우울증상률을 평가한 바 있고, 격년도로 자살생각률을 조사하고 있다. 2009년의 안산 단원 및 상록의 연령표준화된 우울증상률은 각각 4.3%, 4.8%였으며, 경기도의 비교 지역에 속하는 군포, 수원 팔달, 구리, 남양주는 각각 6.2%, 8.3%, 7.1%, 5.9%, 진도 및 해남의 경우 1.7%, 3.5%였다 [20]. 이에 비해 본 조사에서 수집한 안산 지역의 2014년 우울증상률은 2009년에 비해 상당히 높아졌으며, 진도-해남에서도 상승을 보였다. 5년이라는 시간적 간격이 있기 때문에 세월호 사고 때문이라고 단정할 수는 없지만, 일부 영향을 미쳤다고 추정할 수 있다.

자살생각의 경우 2013년에 연령표준화된 증상률이 안산 단원, 상록에서 각각 9.6%, 17.2%였으며, 경기도의 군포, 수원 팔달, 구리, 남양주는 각각 7.2%, 10.6%, 8.1%, 13.5%, 진도 및 해남에서 3.3%, 4.9%로 [20], 1년 만에 남양주를 제외한 전 지역에서 상승을 보였으며, 안산 단원지역의 경우 상승의 절대치가 7.6% 포인트로 가장 높았다. 하지만 안산 지역의 기존 증상률도 타 지역에 비해 높은 편임을 감안해야 하며, 기존 질환자와 취약했던 대상자들이 사고에 대한 경험을 통해 더욱 부정적인 영향을 받았을 가능성도 있으므로 [21] 지속적인 모니터링이 요구된다.

이외에 시계열적으로 비교 가능한 지표로는 지역사회건강조사에서 매년 조사하고 있는 1문항씩의 우울감 및 스트레스에 관한 질문이 있다. '최근 1년 동안 연속적으로 2주 이상 일상생활에 지장이 있을 정도로 슬프거나 절망감 등을 느낀 적이 있습니까?' 에 '예'로 답하는 경우를 우울감 인지율

로, '평소 일상생활 중에 스트레스를 어느 정도로 느끼고 있습니까?' 에 '대단히 많이/많이' 느끼는 경우를 스트레스 인지율로 발표하고 있다. 연령표준화된 우울감 인지율은 안산단원에서 2013년 9.8%에서 2014년 13.0%로 상승하였으며, 안산상록은 12.7%에서 8.1%로 감소하였으나 경기도 평균인 7.0%에 비해 높았다 [20]. 안산단원의 경우 전국 254개 지역 중에서 가장 높았으며, CES-D를 사용한 본 조사 결과와도 비슷한 결과를 보였기에 세월호 사건의 영향이 있었을 것이라 추정 가능하였다. 단 안산 지역의 우울감 인지율은 2013년에도 전국에 비해 75%분위수 이상에 속하는 높은 지역이었다는 점을 감안할 필요가 있다. 스트레스 인지율의 경우 안산단원의 2013년 33.3%에서 31.7%로, 안산상록은 35.6%에서 26.2%로 예년에 비해 다소 줄어들었고, 경기도 평균인 30.2% (95% CI: 29.7-30.7%) 보다는 높거나 낮아 [20] 본 연구에서 BEPSI로 측정한 스트레스와는 다소 다른 양상을 보였다.

본 연구에서는 스트레스나 우울 외에 재난과 관련하여 중요한 영역인 신체 증상 및 불안에 대해서도 평가했는데, 안산 지역의 오즈비가 대조 지역에 비해 높았다. 이러한 결과는 예상하지 못한 재난이 위험에 대한 불확실성을 통해 불안을 발생시키고 [22], 신체의 이상으로 설명되지 않는 여러가지 비특이적 증상을 발생시킨다는 [23] 기존의 연구와 일치했다. 한편, 불안은 단기적으로는 재난에 대한 정상적인 반응이지만, 지속될 경우 쉽게 만성화되고 신체적 정신적인 문제를 야기한다고 알려져 있다 [24].

본 연구에서 나타난 연령별 유병률의 분포는 2009년 지역사회건강조사의 CES-D 결과를 이용, 연령별로 분석한 Oh 등의 연구 [25] 와 유사하여, 세월호 사고와 관련하여 특정 연령대가 두드러진 영향을 받았다는 증거는 관찰되지 않았다. 30대의 우울 유병률이 가장 낮으며, 이후로 연령이 증가함에 따라 유병률이 증가하고, 19-29세는 30대보다 약간 높았다. 단, 본 연구는 성인만을 대상으로 하여 세월호 사건에 심리적 영향을 많이 받았을 것으로 예상되는 청소년 및 어린이들에 대해서는 관찰하지 못하였다. 발달 과정에 있는 미성년자의 심리적 손상은 조기 발견 및 중재가 매우 중요하므로 [26] 추후 이에 대한 연구가 필요할 것으로 보인다.

대형 재난과 관련한 국내의 정신보건학적 연구는 대부분 사건 당사자나 유가족을 대상으로 하며, 본 연구와 같이 지역사회의 일반 인구를 대상으로 한 연구는 많지 않다. 2007년 허베이 스피리트

호 침몰 사고로 인한 해양 오염과 관련해서는 일부 지역사회 기반 연구들이 존재하나, 대부분의 참여자가 방제 작업에 참여 경험이 있거나, 경제적인 피해를 입은 주민들이었다 [8, 9, 27]. 해외의 경우에도 태풍이나 지진 등으로 인한 자연재난과 관련하여 비교적 실질적인 피해를 목격하거나 잠재적인 영향권 내에 있었던 주민들을 대상으로 한 연구가 대부분이다 [5, 28]. 반면 세월호 사고의 경우 지역사회의 물리적 영역 내에서 발생하지 않았고, 주민들의 사건에 대한 경험이 매스컴이나 주변인을 통해 간접적으로 발생하였음에도 불구하고, 해당 지역사회의 청소년들이 주 희생자가 됨으로서 모든 연령층의 지역사회 구성원에게 상당한 정서적 충격을 안겼다는 특이점이 있다. 이런 특징 및 본 연구 결과가 시사하는 바로 볼 때, 재난의 간접적인 영향권에 있었던 지역사회 주민들에 대한 포괄적인 정신보건학적인 모니터링 및 중재의 필요성이 관찰된다.

재난으로 인한 심리적 정서적 긴장은 지역사회 공동체의 긴장감을 고조시키며, 또 다른 재난을 우려하고 자신에게도 그러한 일이 발생할 것을 염려하는 위기감이 증폭된다. 재난을 겪은 이후 변화된 환경과 인적 경제적 손실 등은 장기적인 스트레스로 작용하며 [29], 재난 상황에 대한 정확한 정보가 없어서 발생하는 불필요한 오해나 루머도 지역사회의 회복을 저해하는 요인이 된다. 대부분의 경우에는 심리적 손실이 시간 경과에 따라 완화되지만, 방치하거나 다른 부정적인 요인이 결합되는 경우 장기적인 문제로 발전할 수 있으므로, 적절한 시점의 개입이 필요하다. 본 연구는 재난 후 약 4-6개월 시점에 이루어졌는데, 동일한 시점에서 이루어진 다른 연구들에서도 높은 우울증과 외상후 스트레스 등이 관찰되었다는 연구 결과가 있으며 [28], 일부에서는 수년 후에도 지속되었다 [5]. 9/11테러와 관련한 한 추적 조사에서, 장기적인 정신적 증상 지속은 단순히 재난에 직접적으로 노출되었는지의 여부보다는 재난 직후 적절한 시점에 조치를 받았는지와 관련이 있었으며 [2], 다른 연구에서는 지진 피해 이후 7년이 지난 후에도 높은 신체증상, 우울 및 불안 등이 관찰되었다는 보고가 있었다 [29].

본 연구 결과에는 몇 가지 한계점이 존재하는데, 중요한 한계로 적절한 대조군을 확보하기 힘들었다는 점이 있었다. 지역적 편차를 최대한 배제하기 위해 안산 지역과 같은 도에 속하는 경기도에서 4개 지역을 선정하여 대조군으로 사용하였는데, 경기도 내에서도 각 지역의 편차가 존재하여 인구사회학적 특성을 보정하였다 하더라도 제한점이 있었다. 본 조사 결과 안산 지역의 유병

률이 높았지만 기존 조사에서도 정신건강 분야와 관련된 보건 지표가 다소 높은 지역에 속했다. 비슷한 이유로, 재난이 발생한 지역인 진도 및 해남은 과거에도 타 지역에 비해 유병률이 낮았고 경기도와는 지역적 특성이 구별되어 본 조사 대상 지역 중 비교성을 가진 대조군이 없었다. 또한 세월호 사건은 전국적 비극으로서 매스컴 등을 통해 실시간으로 전파되면서 재난의 심리적 영향이 전국적으로 발생하였다. 그로 인해 지역간 비교를 하였을 경우 사건 지역에 발생한 재난의 영향이 과소평가될 수 있었다.

또 다른 한계로 기존의 자료가 부족하여, 시계열적인 변화를 관찰하기는 불가능하였다. 하지만 다른 형태의 변수 및 과거의 자료를 이용하여 추정한 결과 상당한 의미를 관찰할 수 있었다. 단, 우울증상률과 자살 생각의 경우 지역사회건강조사의 경우 조사원이 인터뷰 방식으로 조사하는 반면 본 연구에서는 자기기입식으로 조사하였기 때문에 같은 도구를 사용하였더라도 민감한 질문에 대해 더욱 솔직하게 답하였을 가능성이 있고, 이에 따라 과거의 유병률이 과소평가되었을 가능성을 배제할 수 없었다.

또한 지역 단위의 표본 추출을 통한 조사 체계의 특성 상 일반 지역사회 주민 중 유가족이나 친척 등 세월호와의 직접적 관련이 있는 대상자를 따로 표집하지 않았고, 사건과 관련된 선입견을 배제하기 위해 세월호 사건과의 직접적 관련성을 평가하는 문항을 포함시키지 않았기 때문에 재난의 노출 정도에 따른 세부 위험 분류 모델을 적용할 수 없는 한계가 있었다는 것은 이 연구의 아쉬운 부분이었다. 또한, 사건을 직접적으로 경험하지 않은 지역사회의 표본이 대부분이었기 때문에, 재난 관련 건강영향 평가 시 중요한 영역인 외상 후 스트레스 관련 조사 척도를 적용할 수 없었다. 하지만 국가적 재난 발생 시 사건의 간접 경험을 통해 직접적인 피해자가 아닌 주민들에게도 외상 후 스트레스 가 발생하였다는 여러 연구들이 있다 [3]. 일반 인구를 대상으로 사건 관련 스트레스를 평가할 수 있는 선별 도구의 개발이 필요할 것으로 보인다.

이러한 한계에도 불구하고 기존에 유용성이 인정된 선별검사 도구를 사용하여 다양한 측면의 정신건강 영역에 대해 평가하였고, 기존 지역건강조사체계를 이용하여 체계적으로 추출된 표본을 사용하여 각 지역의 정신건강 수준을 대표성 있게 평가했다는 점은 본 연구의 큰 강점이다. 또한, 무엇보다도 시기적으로 재난 이후 6개월 이내의 정신건강 수준을 평가할 수 있었다는 점에 시사

점이 크다. 본 연구를 통한 단면적인 조사 결과를 고려했을 때, 재난 피해 지역의 정신건강 회복을 위한 적극적인 공중보건학적 개입이 필요하다고 판단된다. 또한 추가 연구를 통해 지역사회의 변화 및 회복 과정을 관찰하는 것이 학문적으로도, 사회적으로도 큰 의미가 있을 것이다.

REFERENCES

1.Galea S, Ahern J, Resnick H, Kilpatrick D, Bucuvalas M, Gold J, et al. Psychological sequelae of the September 11 terrorist attacks in New York City. New England Journal of Medicine 2002;346:982-987.

2.Silver RC, Holman EA, McIntosh DN, Poulin M, Gil-Rivas V. Nationwide longitudinal study of psychological responses to September 11. JAMA 2002;288:1235-1244.

3.Galea S. Trends of Probable Post-Traumatic Stress Disorder in New York City after the September 11 Terrorist Attacks. Am J Epidemiol 2003;158:514-524.

4.Schlenger WE, Caddell JM, Ebert L, Jordan BK, Rourke KM, Wilson D, et al. Psychological reactions to terrorist attacks: findings from the National Study of Americans' Reactions to September 11. JAMA 2002;288:581-588.

5.Kessler RC, Galea S, Gruber MJ, Sampson NA, Ursano RJ, Wessely S. Trends in mental illness and suicidality after Hurricane Katrina. Mol Psychiatry 2008;13:374-384.

6.Lee DY, Na J, Sim M. Psychological Reactions and Physical Trauma by Types of Disasters: View from Man-Made Disaster. J Korean Neuropsychiatr Assoc 2015;54:261.

7.Norris FH, Friedman MJ, Watson PJ, Byrne CM, Diaz E, Kaniasty K. 60,000 disaster victims speak: Part I. An empirical review of the empirical literature, 1981—2001. Psychiatry 2002;65:207-239.

8.Song M, Hong YC, Cheong HK, Ha M, Kwon H, Ha EH, et al. Psychological health in residents participating in clean-up works of Hebei Spirit oil spill. J Prev Med Public Health 2009;42:82-88.

9.Kim Y-M, Park J-H, Choi K, Noh SR, Choi Y-H, Cheong H-K. Burden of disease attributable to the Hebei Spirit oil spill in Taean, Korea. BMJ open 2013;3:e003334.

10.Federal Emergency Management Agency (FEMA) (2013) Crisis counseling assistance and training program guidance. CCP Application Toolkit, Version 4.0 US Department of Health and Human Services.
http://www.dhhr.wv.gov/healthprep/programs/behavioralhealth/Documents/FEMA%20CCP%20Toolkit.pdf.

11.Gwon MC. First Report About Actual Condition of Sewol Ferry Victims' Families [homepage on the Internet]. Seoul: CBS Nocut news; 2015 [updated 2015 Apr 6; cited 2015 Oct 12]. Available from: http://www.nocutnews.co.kr/news/4393074.

12.Kim YT, Choi BY, Lee KO, Kim H, Chun JH, Kim SY, et al. Overview of Korean Community Health Survey [in Korean]. J Korean Med Assoc 2012;55:74.

13.Radloff LS. The CES-D scale a self-report depression scale for research in the general population. Appl Psychol Meas 1977;1:385-401.

14.Cho MJ, Kim KH. Diagnostic validity of the CES-D (Korean version) in the assessment of DSM-III-R major depression. J Korean Neuropsychiatr Assoc 1993;32:381-399.

15.Frank SH, Zyzanski SJ. Stress in the clinical setting: the Brief Encounter Psychosocial Instrument. J Fam Pract 1988:533-539.

16.Yim J, Bae J, Choi S, Kim S, Hwang H, Huh B. The validity of modified Korean-translated BEPSI (Brief Encounter Psychosocial Instrument) as instrument of stress measurement in outpatient clinic.

J Korean Acad Fam Med 1996;17:42-53.
17.Kroenke K, Spitzer RL, Williams JB. The PHQ-15: validity of a new measure for evaluating the severity of somatic symptoms. Psychosom Med 2002;64:258-266.
18.Han C, Pae C-U, Patkar AA, Masand PS, Kim KW, Joe S-H, et al. Psychometric Properties of the Patient Health Questionnaire–15 (PHQ–15) for Measuring the Somatic Symptoms of Psychiatric Outpatients. Psychosomatics 2009;50:580-585.
19.Spitzer RL, Kroenke K, Williams JB, Löwe B. A brief measure for assessing generalized anxiety disorder: the GAD-7. Arch Intern Med 2006;166:1092-1097.
20.Korea Centers for Disease Control and Prevention. Community Health Survey. [cited 2015 Oct 12]. Available from: https://chs.cdc.go.kr
21.Nolen-Hoeksema S, Morrow J. A Prospective Study of Depression and Posttraumatic Stress Symptoms After a Natural Disaster: The 1989 Loma Prieta Earthquake. J Pers Soc Psychol 1991;61:115-121.
22.Norris FH, Stevens SP, Pfefferbaum B, Wyche KF, Pfefferbaum RL. Community resilience as a metaphor, theory, set of capacities, and strategy for disaster readiness. Am J Community Psychol 2008;41:127-150.
23.Escobar JI, Canino G, Rubio-Stipec M, Bravo M. Somatic symptoms after a natural disaster: A prospective study. Am J Psychiatry 1992;149:965-967.
24.Norris FH, Stevens SP. Community Resilience and the Principles of Mass Trauma Intervention. Psychiatry: Interpersonal and Biological Processes 2007;70:320-328.
25.Oh DH, Kim SA, Lee HY, Seo JY, Choi BY, Nam JH. Prevalence and correlates of depressive symptoms in korean adults: results of a 2009 korean community health survey. J Korean Med Sci 2013;28:128-135.
26.Reijneveld SA, Crone MR, Verhulst FC, Verloove-Vanhorick SP. The effect of a severe disaster on the mental health of adolescents: a controlled study. The Lancet 2003;362:691-696.
27.Eum JH, Cheong HK, Ha M, Kwon HJ, Ha EH, Hong YC, et al. Hebei Spirit oil spill exposure and acute neuropsychiatric effects on residents participating in clean-up work. Korean J Epidemiol 2008;30:239-251.
28.Chou FHC, Su TTP, Chou P, Ou-Yang WC, Lu MK, Chien IC. Survey of psychiatric disorders in a Taiwanese village population six months after a major earthquake. J Formos Med Assoc 2005;104:308-317.
29.Bland SH, O'Leary ES, Farinaro E, Jossa F, Trevisan M. Long-term psychological effects of natural disasters. Psychosom Med 1996;58:18-24.
