# Supplementary material for: Community mental health status six months after the Sewol ferry disaster in Ansan, Korea
Source: Epidemiol Health. 2015 Oct 23;37:e2015046. doi: 10.4178/epih/e2015046 (PMC4835706; doi:10.4178/epih/e2015046)
Supplement: Supplementary file 2 [file epih-37-e2015046-Appendix.pdf]

Epidemiology and Health 2015;37:e2015046

Appendix 1.

지역사회 건강조사 기반 사회심리 및 안전인식도 조사

조 · 사 · 원 : · ____________________

◈ 아래의 칸은 조사원이 기입하는 부분입니다.

일자		관리번호	시도	보건소			표본지점					가구				가구원	

◈ 아래의 칸은 책임대학에서 기입하는 부분입니다.

	표본지점					가구				가구원	
	⓪	⓪	⓪	⓪	⓪	⓪	⓪	⓪	⓪	⓪	⓪
① 군포시보건소	①	①	①	①	①	①	①	①	①	①	①
② 수원시 팔달구보건소	②	②	②	②	②	②	②	②	②		②
③ 안산시 단원보건소	③	③	③	③	③	③	③	③	③		③
④ 안산시 상록수보건소	④	④	④	④	④	④	④	④	④		④
⑤ 남양주시보건소	⑤	⑤	⑤	⑤	⑤	⑤	⑤	⑤	⑤		⑤
⑥ 구리시보건소	⑥	⑥	⑥	⑥	⑥	⑥	⑥	⑥	⑥		⑥
⑦ 진도군보건소	⑦	⑦	⑦	⑦	⑦	⑦	⑦	⑦	⑦		⑦
⑧ 해남군보건소	⑧	⑧	⑧	⑧	⑧	⑧	⑧	⑧	⑧		⑧
	⑨	⑨	⑨	⑨	⑨	⑨	⑨	⑨	⑨		⑨

지역사회 건강조사 기반
사회심리 및 안전인식도 조사
〈자기기입식〉

※ 다음 문항을 읽고, 해당되는 "□"를 → "■"로 까맣게 칠해주세요 ※
컴퓨터로 자동 입력되는 용지이오니 반드시 위와 같이 표기하여 주시기 바랍니다.

Ⅰ. **지난 4주 동안** 다음 신체 증상 때문에 얼마나 자주 방해를 받았습니까?

항 목	전혀 시달리지 않음	약간 시달림	대단히 시달림
1. 위통	□	□	□
2. 허리 통증	□	□	□
3. 팔, 다리, 관절 (무릎, 고관절 등)의 통증	□	□	□
4. 생리기간 동안 생리통 등의 문제 **[여성만 응답함]**	□	□	□
5. 두통	□	□	□
6. 가슴 통증, 흉통	□	□	□
7. 어지러움	□	□	□
8. 기절할 것 같음	□	□	□
9. 심장이 빨리 뜀	□	□	□
10. 숨이 참	□	□	□
11. 성교 통증 등의 문제	□	□	□
12. 변비, 묽은 변이나 설사	□	□	□
13. 메슥거림, 방귀, 소화불량	□	□	□
14. 피로감, 기운 없음	□	□	□
15. 수면의 어려움	□	□	□

※ 다음 문항을 읽고, 해당되는 "□"를 → "■"로 까맣게 칠해주세요 ※
컴퓨터로 자동 입력되는 용지이오니 반드시 위와 같이 표기하여 주시기 바랍니다.

Ⅱ. 다음 각 항목에 대한 귀하의 생각은 어떻습니까?

항 목	예	아니오
1. 우리 동네 사람들은 서로 믿고 신뢰할 수 있다	□	□
2. 이웃에 경조사가 있을 때, 주민 사이에 서로 도움을 주고받는 전통이 있다	□	□
3. 우리 동네의 전반적 안전수준(자연재해, 교통사고, 농작업 사고, 범죄)에 대해 만족한다	□	□
4. 우리 동네의 자연환경(공기질, 수질 등)에 대해 만족한다	□	□
5. 우리 동네의 생활환경(전기, 상하수도, 쓰레기 수거, 스포츠시설 등)에 대해 만족한다	□	□
6. 우리 동네의 대중교통 여건(버스, 택시, 지하철, 기차 등)에 대해 만족한다	□	□
7. 우리 동네의 의료서비스 여건(보건소, 병의원, 한방병의원, 약국 등)에 대해 만족한다	□	□

Ⅲ. 다음 각 분야마다 우리 사회가 어느 정도 안전하다고 생각하십니까?

분 야	매우 안전	비교적 안전	보통	비교적 불안	매우 불안
1. 국가 안보(전쟁 가능성, 북핵문제 등)	□	□	□	□	□
2. 자연재해(태풍, 지진 등)	□	□	□	□	□
3. 건축물 및 시설물(주택, 교량 등)	□	□	□	□	□
4. 교통사고	□	□	□	□	□
5. 화재(산불 포함)	□	□	□	□	□
6. 식량 안보(곡물가 폭등, 식량 부족 등)	□	□	□	□	□
7. 정보 보안(컴퓨터바이러스, 기타 해킹 등)	□	□	□	□	□
8. 신종 전염병(신종 바이러스, 조류독감, 사스 등)	□	□	□	□	□
9. 범죄 위험	□	□	□	□	□
10. 전반적인 사회 안전	□	□	□	□	□

※ 다음 문항을 읽고, 해당되는 "□"를 → "■"로 까맣게 칠해주세요 ※
컴퓨터로 자동 입력되는 용지이오니 반드시 위와 같이 표기하여 주시기 바랍니다.

Ⅳ. 지난 1주일 동안 다음과 같은 일들이 얼마나 자주 일어났습니까?

항 목	1일 미만	1~2 일	3~4 일	5일 이상
1. 평소에는 아무렇지도 않던 일들이 괴롭고 귀찮게 느껴졌다	□	□	□	□
2. 먹고 싶지 않고 식욕이 없었다	□	□	□	□
3. 어느 누가 도와준다 하더라도 나의 울적한 기분을 떨쳐 버릴 수 없을 것 같았다	□	□	□	□
4. 무슨 일을 하든 정신을 집중하기가 힘들었다	□	□	□	□
5. 비교적 잘 지냈다	□	□	□	□
6. 상당히 우울했다	□	□	□	□
7. 모든 일들이 힘들게 느껴졌다	□	□	□	□
8. 앞일이 암담하게 느껴졌다	□	□	□	□
9. 지금까지의 내 인생은 실패작이라는 생각이 들었다	□	□	□	□
10. 적어도 보통 사람들만큼의 능력은 있다고 생각했다	□	□	□	□
11. 잠을 설쳤다(잠을 잘 이루지 못했다)	□	□	□	□
12. 두려움을 느꼈다	□	□	□	□
13. 평소에 비해 말수가 적었다	□	□	□	□
14. 세상에 홀로 있는 듯한 외로움을 느꼈다	□	□	□	□
15. 큰 불만 없이 생활했다	□	□	□	□
16. 사람들이 나에게 차갑게 대하는 것 같았다	□	□	□	□
17. 갑자기 울음이 나왔다	□	□	□	□
18. 마음이 슬펐다	□	□	□	□
19. 사람들이 나를 싫어하는 것 같았다	□	□	□	□
20. 도무지 뭘 해 나갈 엄두가 나지 않았다	□	□	□	□

Ⅴ. 지난 1년 동안 다음과 같은 일들이 있었습니까?

항 목	예	아니오
1. 최근 1년 동안 죽고 싶다는 생각을 해 본 적이 있습니까?	□	□
2. 최근 1년 동안 실제로 자살시도를 해 본 적이 있습니까?	□	□

2014 지역사회건강조사 4

※ 다음 문항을 읽고, 해당되는 "□"를 → "■"로 까맣게 칠해주세요 ※

컴퓨터로 자동 입력되는 용지이오니 반드시 위와 같이 표기하여 주시기 바랍니다.

Ⅵ. 지난 1달 동안 다음과 같은 일들이 얼마나 있었습니까?

항 목	전혀 없었다	간혹 있었다	종종 여러번 있었다	거의 언제나 있었다	항상 있었다
1. 살아가는데, 정신적·신체적으로 감당하기 힘들다고 느낀적이 있습니까?	□	□	□	□	□
2. 자신의 생활 신념에 따라 살려고 애쓰다가 좌절을 느낀적이 있습니까?	□	□	□	□	□
3. 한 인간으로서 기본적인 요구가 충족되지 않았다고 느낀적이 있습니까?	□	□	□	□	□
4. 미래에 대해 불확실하게 느끼거나 불안해 한 적이 있습니까?	□	□	□	□	□
5. 할 일이 너무 많아 정말 중요한 일들을 잊은 적이 있습니까?	□	□	□	□	□

Ⅶ. 지난 2주 동안, 다음의 문제들로 인해서 얼마나 자주 방해를 받았습니까?

항 목	전혀 방해 받지 않았다	며칠 동안 방해 받았다	절반 이상 방해 받았다	거의 매일 방해 받았다
1. 초조하거나 불안하거나 조마조마하게 느낀다	□	□	□	□
2. 걱정하는 것을 멈추거나 조절할 수가 없다	□	□	□	□
3. 여러 가지 것들에 대해 걱정을 너무 많이 한다	□	□	□	□
4. 편하게 있기가 어렵다	□	□	□	□
5. 너무 안절부절못해서 가만히 있기가 힘들다	□	□	□	□
6. 쉽게 짜증이 나거나 쉽게 성을 내게 된다	□	□	□	□
7. 마치 끔찍한 일이 생길 것처럼 두렵게 느껴진다	□	□	□	□

Ⅷ. 모든 사항을 고려할 때, 최근 귀하의 삶에 (대체로) 어느 정도 만족합니까? 해당되는 "□"를 → "■"로 칠해주세요.

1	2	3	4	5	6	7	8	9	10
□	□	□	□	□	□	□	□	□	□

매우 불만족 ... 매우 만족

*** 설문에 응답해 주셔서 대단히 감사합니다 ***
